# A Machine Learning Approach to Simulate Gene Expression and Infer Gene Regulatory Networks †

**DOI:** 10.3390/e25081214

**Published:** 2023-08-15

**Authors:** Francesco Zito, Vincenzo Cutello, Mario Pavone

**Affiliations:** Department of Mathematics and Computer Science, University of Catania, 95125 Catania, Italy

**Keywords:** reverse engineering, gene regulatory network, machine learning, time-series forecasting, metaheuristic, complex network

## Abstract

The ability to simulate gene expression and infer gene regulatory networks has vast potential applications in various fields, including medicine, agriculture, and environmental science. In recent years, machine learning approaches to simulate gene expression and infer gene regulatory networks have gained significant attention as a promising area of research. By simulating gene expression, we can gain insights into the complex mechanisms that control gene expression and how they are affected by various environmental factors. This knowledge can be used to develop new treatments for genetic diseases, improve crop yields, and better understand the evolution of species. In this article, we address this issue by focusing on a novel method capable of simulating the gene expression regulation of a group of genes and their mutual interactions. Our framework enables us to simulate the regulation of gene expression in response to alterations or perturbations that can affect the expression of a gene. We use both artificial and real benchmarks to empirically evaluate the effectiveness of our methodology. Furthermore, we compare our method with existing ones to understand its advantages and disadvantages. We also present future ideas for improvement to enhance the effectiveness of our method. Overall, our approach has the potential to greatly improve the field of gene expression simulation and gene regulatory network inference, possibly leading to significant advancements in genetics.

## 1. Introduction

Understanding the intrinsic relationship between genes with the aim of treating known diseases is currently one of the great challenges in genetics [1]. Although this topic may seem to be only a biological problem, it actually involves many areas of computer science. Due to the complexity of this problem, traditional mathematical methods such as Ordinary Differential Equations (ODEs), which rely on estimates of gene expression levels over time through a continuous model, may be inaccurate for a larger number of genes and require high-quality data to create an acceptable model [2]. Machine-learning-based techniques have emerged as a promising approach for gene regulatory network inference, outperforming other methods based on mutual information [3]. These techniques can be broadly classified into two categories. The first category involves using observations to create a model that approximates the real system, which is then used to construct a complex network that identifies the regulatory genes for other genes, known as the gene regulatory network [4]. The second category involves the direct creation of a gene regulatory network through observations, without the need to estimate a model representing the dynamics of gene expression [3,5].

In this research work, we focus on the first category. The whole process of creating a gene regulatory network is split into three phases: (1) creation of a model capable of approximating the actual behavior of genes, as well as their interaction; (2) inference of the corresponding gene regulatory network showing the relationships between genes; (3) evaluation of the resulting gene regulatory network against benchmarks. This manuscript aims to analyze each phase in detail, focusing in particular on the first step, which is the main innovation of our approach. In our previous work [6], we addressed the problem of inferring a gene regulatory network with a reverse engineering approach. The idea is to build an artificial environmental setting capable of replicating the behavior of a real case based solely on the observation of the variables of interest and to generate a complex network that reveals the relationships among the variables in the system. In the gene regulation problem, each variable represents the expression level of a gene that evolves over time. By observing how the gene expression of a group of genes changes over time, it is possible to train an artificial-neural-network-based model. In the present work, we aim to extend and improve our model by addressing some of its weaknesses and to test it on a different gene expression dataset such as the DREAM4 Challenge [7] and SOS DNA Repair [8].

### 1.1. Related Work

Before the spread of machine learning in the field of genetics, Boolean networks were generally used to describe gene regulatory networks. All biological components can be described by binary states and their interactions by Boolean functions [9]. Boolean networks are relatively simple to implement, but their implementation requires noise-free, discrete data, which can be difficult to obtain when working with real-world data [10].

In recent years, several methods to extract a gene regulatory network have been presented. In [11], the authors divided the methods for inferring a gene regulatory network from gene expression data into three main groups: (*i*) model-based methods; (*ii*) information-theory-based methods; and (*iii*) machine learning methods. Some experimental tests have shown that machine learning methods can obtain a high accuracy in predicting gene interactions [12]. One approach to inferring a gene regulatory network from a gene expression dataset is to use differential equations. This requires a mathematical model of the changes in gene expression over time using ordinary differential equations, which can provide insight into the underlying dynamics of the system. By analyzing the behavior of these equations, one can gain a better understanding of how genes interact and regulate each other within the network [13]. The difficulty in such an approach is, clearly, to build a differential equation model from data. To this end, several methods have been proposed in the literature. An example can be found in [14], where a metaheuristic was used to find the parameters of an *S-system model* describing the dynamics of gene expression. Another example can be found in [15], where a complex-valued ordinary differential equation model was created using genetic programming. In addition, it is possible to directly predict the interaction between genes using a gene expression dataset. One method used in this field is GENIE3 presented in [16] and its improvement called DynamicGENIE3 presented in [17]. An improvement on the previously cited method in this category can be found in [18], where different inference methods are combined to increase the accuracy of the resulting gene regulatory network.

Rather than using a specific strategy to predict each arc of a gene regulatory network, an alternative approach involves the construction of a comprehensive network that assumes all possible interactions between genes, represented as a strongly connected graph, and subsequently applying a pruning strategy to eliminate non-corresponding arcs. One example of such a method, which employs an information–theoretic algorithm, is described in [19].

### 1.2. Contributions

In light of the promising results of machine-learning-based methods for predicting genetic interactions, in this article, we propose a general methodology based on machine learning. Our goal is to lay the foundation for a reverse engineering approach to figure out genetic interactions. However, unlike the works mentioned above, we are not so much interested in providing a specific method to directly infer a gene regulatory network, but rather in providing a reverse engineering framework to replicate the actual behavior of genes based on their observations and consequently understand their interactions. Within such a framework, biologists or researchers in general can test hypotheses about how genes function and how they respond to different stimuli, without having to conduct expensive and time-consuming experiments in the lab. This can lead to a better understanding of how genes work and how they can be manipulated to treat diseases and improve human health.

### 1.3. Outline of the Present Work

In Section 2, we introduce the meaning of gene expression and the used terminologies. Our focus is not so much on the biological aspect, but rather on providing readers with basic knowledge to understand the application domain in which our reverse engineering framework is used. In particular, in Section 2.3, we discuss the datasets we used to validate our methodology. In Section 3, we introduce and discuss our artificial environmental setting and its entities used to replicate gene behavior. In Section 4, we describe the procedure for creating such an artificial environmental setting from gene expression data. We provide results and further analysis in Section 5. Finally, in Section 6, we present our conclusions and proposals for future work.

## 2. Background

The process by which the instructions in our *DNA* are transformed into a functioning product, such as a protein, is known as *gene expression* [20]. Gene expression allows a cell to respond to changes in its environment. The *regulation of gene expression* (or just *gene regulation*) is a very complex process that takes into account several biological factors to respond, for example, to environmental stimuli or to adapt to new food sources [21,22]. Gene regulation involves a variety of mechanisms used by cells to increase or decrease the production of certain gene products. Thus, it functions like an on/off switch that regulates the amount of proteins produced.

Considering the huge amount of gene products that are present in a multicellular organism, the regulatory mechanisms are represented in a directed graph, called the *regulatory network*, to help better understand the regulatory mechanisms. A regulatory network reveals the interactions between genes, proteins, mRNAs, and cellular processes and provides important information about the development of diseases [23]. Knowledge of a regulatory network for an entire organism or for a small group of genes is crucial for a full understanding of the life process of an organism and how gene products interact with each other [24]. Once this is clear, it is possible to send external chemical signals to inhibit a gene that could be dangerous to the life of an organism, such as the development of a cancer cell or a genetic disease [25].

### 2.1. Gene Regulatory Network

A gene regulatory network is a directed graph where the nodes represent genes, and the directed arcs model the interactions between the genes [26]. Specifically, a *Gene Regulatory Network* (GRN) represents the regulatory process of gene expression in an organism. An arc between two nodes, i.e., genes, mainly provides information about the regulatory process. In the context of inferring gene regulatory networks, the presence of a direct arc from gene $G_{i}$ to gene $G_{j}$ indicates that $G_{i}$ is a regulatory gene, also known as a regulator [27]. This implies that any alteration in the expression of $G_{i}$ will have a consequential impact on the expression of $G_{j}$, according to the principle of cause and effect. In other words, the regulatory gene $G_{i}$ is capable of influencing the expression of its target gene $G_{j}$, thereby establishing a cause-and-effect relationship between the two genes.

**Figure 1:**
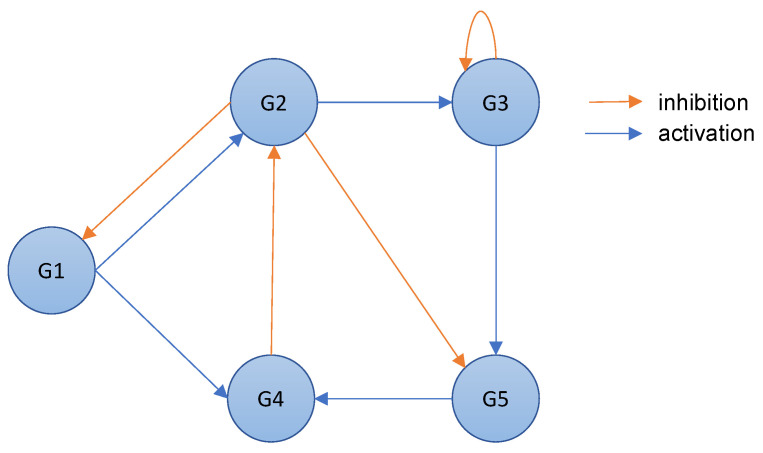
An example of a gene regulatory network that includes gene regulation information.

A gene regulatory network can, therefore, combine more-detailed regulatory information. In fact, a regulatory gene controls the expression of its associated genes in a positive or negative way. When the expression level of the regulator reaches a threshold, another gene can be activated or inhibited based on that level [28]. This results in a change in the expression level of the regulated gene: if the gene expression decreases, the gene is inhibited; otherwise, it is activated. Figure 1 shows an example of a gene regulatory network. As can be seen, there are two types of arcs in a gene regulatory network: activation arcs and inhibition arcs.

### 2.2. Inferring a Gene Regulatory Network

**Figure 2:**
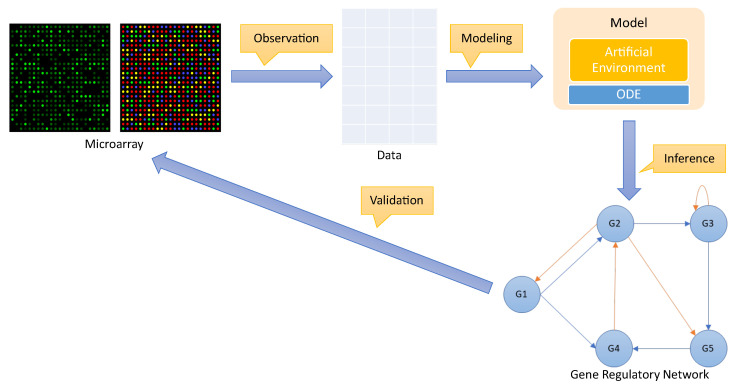
Process to infer a gene regulatory network.

The process of inferring a gene regulatory network for a cellular organism can be divided into four distinct phases, which we label: observation; modeling; inference; and validation. The whole process is shown in Figure 2.

1. **Observation:** The first step is to observe how the gene expression of a group of genes responds to external perturbations in a real organism. This can be performed using various strategies, such as microarray technology [29]. The level of gene expression for each gene is recorded over time to create a time-series dataset containing gene expression for the genes under observation. Typically, such a dataset is represented as a matrix $D\in R^{M\times N}$, where *N* is the number of genes and *M* is the number of observations for each gene over time.
2. **Modeling:** The gene expression time-series dataset is used to train a model that can be based on differential equations [30] or design an artificial environmental setting, which we will introduce in Section 3.
3. **Inference:** The model created in the previous phase is used to make predictions about the relationships between genes in order to discover regulatory genes. This information can, therefore, be used to draw a complex network, i.e., a gene regulatory network, showing these relationships.
4. **Validation:** Finally, to validate the accuracy of a predicted gene regulatory network, it is essential to compare it with the target network. However, this comparison can only be performed on an artificial dataset where the gene regulatory network is known beforehand. In a real organism, we do not have access to a gene regulatory network, and therefore, the validation of the predicted gene regulatory network must be performed empirically and in the field.

### 2.3. Datasets

There are several datasets that can be used to validate a methodology of inferring a gene regulatory network. These datasets can be broadly categorized into two types: steady-state and time-series datasets. In the steady-state dataset, the expression level of genes is reported in a stable state under different perturbations such as gene knockout, gene knockdown, etc. On the other hand, time-series datasets contain the expression levels of each gene at different time points. Another way to classify gene expression datasets is based on their source, which can be either synthetic or real. Synthetic datasets are generated using software that produces gene expression levels over time while preserving certain properties of genes. For example, GeneNetWeaver [7] is a popular tool used to generate synthetic datasets, such as those used in the DREAM4 Challenge. In contrast, real datasets are obtained by observing the actual gene expression over time and how it responds to perturbation. This can be achieved through the use of technologies, such as microarray technology [24], that allow for the measurement of gene expression levels. To conduct our experiments, we used several datasets, and their sources are summarized in Table 1.

## 3. Modeling

We propose a novel method for modeling the gene expression of group of genes of an organism by machine learning. In the method of ordinary differential equations, as well as in all other methods that follow this approach, distinct equations are formulated for each gene to study its dynamic behavior. If we consider a group of *n* genes whose mutual interaction and degree of correlation we want to estimate, the level of expression of the *i*-th gene, denoted by $x_{i}\left(t\right)$, can be described over time by the following:

(1) $$\frac{\partial x_{i}\left(t\right)}{\partial t}=g_{i}\left(x_{1}\left(t\right),\ldots ,x_{i}\left(t\right),\ldots ,x_{n}\left(t\right),t\right)+K_{i},\;\forall i=\left(1,2,\ldots ,n\right),$$

where $g_{i}\left(\cdot \right)$ is a non-linear function representing the dynamics of the expression level of the *i*-th gene and $K_{i}$ is a constant. It follows that the gene expression level at time *t* is strongly related to the expression level of the other genes. Each gene may have more or less influence on gene *i* depending on how strongly it is correlated. The genes that have a greater influence than others on the expression level of gene *i* are considered regulators according to the definition of the term *regulatory gene* introduced in Section 2.1. If the mathematical expression for $g_{i}\left(\cdot \right)$ were available, it would be possible to identify the regulatory gene directly. However, since this information is not accessible, alternative methods such as metaheuristics [13] or machine learning must be employed, as is the case in our study.

Equation (1) allows us to identify the regulatory genes, but it does not provide information on the actual gene expression levels at different time points. To address this limitation, we propose in our model a modified approach. Rather than directly calculating the derivative of gene expression with respect to time, denoted by $g_{i}\left(\cdot \right)$, we reformulate the equation as follows:

(2) $$x_{i}\left(t\right)=f_{i}\left(x_{1}\left(t\right),\ldots ,x_{i}\left(t\right),\ldots ,x_{n}\left(t\right),t\right),\;\forall i=\left(1,2,\ldots ,n\right),$$

where $x_{i}\left(t\right)$ is the gene expression level of the *i*-th gene at time *t* and $f_{i}\left(\cdot \right)$ is a non-linear function, whose mathematical expression can be obtained from Equation (1), knowing the initial condition, that is:

(3) $$\left\{\begin{array}{c} f_{i}\left(x_{1}\left(t\right),\ldots ,x_{i}\left(t\right),\ldots ,x_{n}\left(t\right),t\right)=\int_{0}^{t}g_{i}\left(x_{1}\left(t\right),\ldots ,x_{i}\left(t\right),\ldots ,x_{n}\left(t\right),t\right)+K_{i}\partial t,\\ x_{i}\left(0\right)=x_{i_{0}}, \end{array}\right.$$

where $x_{i_{0}}$ is the gene expression value of the *i*-th gene at the initial time, which in many cases is zero.

Therefore, the behavior of the *i*-th gene is entirely defined by the function $f_{i}\left(\cdot \right)$. The task of modeling the function $f_{i}\left(\cdot \right)$ for each gene is assigned to an ad hoc unit, denoted by the term *agent*. Each agent can interact with other agents, just like genes, in the so-called artificial environmental setting, which models a real environment. Before examining in detail what an agent and an artificial environmental setting are, it is necessary to point out that we are not looking directly for the mathematical expression of $f_{i}\left(\cdot \right)$, but we are generating as many agents as there are genes. Subsequently, the agents are used to determine the expression levels of the genes over time just as well as if we had used the mathematical expression. To simplify the discussion and since the time dependence is already included in the expression level of the genes, we prefer to remove the direct dependence on it within the function $f_{i}\left(\cdot \right)$ in Equation (2), which is then rewritten as follows:

(4) $$x_{i}\left(t\right)=f_{i}\left(x_{1}\left(t\right),\ldots ,x_{n}\left(t\right)\right),\;\forall i=\left(1,2,\ldots ,n\right).$$

While the function described earlier is continuous in time, practical considerations, due to computational limitations, require us to use a discrete function instead. Thus, we will introduce a new discrete function in the next subsection.

We will now describe three crucial concepts that form the backbone of our methodology: the agent, the artificial environmental setting, and the simulation of gene expression. By understanding these concepts, we can gain a deeper insight into the workings of our approach and how it can be applied to various domains.

### 3.1. Agent

**Figure 3:**
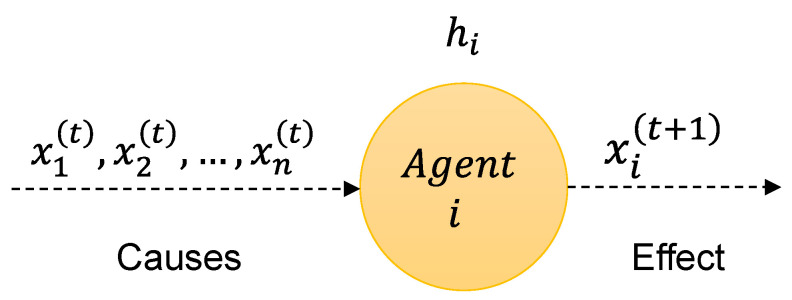
Visual representation of an agent.

As mentioned in the preceding paragraph, agents play a crucial role in replicating gene behavior. They determine whether a gene’s expression should be increased or decreased based on the expression of other genes, which are themselves regulated by other agents. Each agent is responsible for regulating the expression of a single gene. We will use the notation $A_{i}$ to refer to the agent responsible for regulating the *i*-th gene. Specifically, an agent $A_{i}$ acts as a predictor, forecasting the level of gene expression at time *t* based on the expression levels of other genes at time t−1. This behavior can be formally modeled by a function $h_{i}:R^{n}\to R$, which, given the expression value of all genes at time *t*, returns the expression value of gene *i* at time t+1. Such a behavior can be seen as a consequence of the principle of a causal relationship: when an agent receives as the input the expression level of a gene (cause), it regulates the expression level of its gene (effect). An agent $A_{i}$ can be formally defined as:

(5) $$A_{i}:\;x_{i}^{\left(t+1\right)}=h_{i}\left(x_{1}^{\left(t\right)},\ldots ,x_{n}^{\left(t\right)}\right),$$

where the notation $x_{i}^{\left(t\right)}$ represents the expression level of the *i*-th gene at time step *t* (Figure 3).

A formulation of Equation (5) was presented in our earlier paper [6]. However, in our experiments, we found that this formulation does not take into account the trend of a gene expression level over time. Theoretically, an agent should regulate the level of gene expression of a gene at time t+1 by taking into account not only what happened at the previous time *t*, but also some kind of history of gene expression from the initial time to *t*. Practically, it follows that we have modified Equation (5) to accomplish this behavior.

Supposing that *m* indicates the number of time steps (m≥1) and an agent is considering predicting the gene expression at time t+1, the agent function can be redefined as $h_{i}:R^{n\times m}\to R$, that is:

(6) $$A_{i}:x_{i}^{\left(t+1\right)}=h_{i}\left(X^{\left(t\right)}\right),$$

where $X^{\left(t\right)}\in R^{n\times m}$ is a matrix containing the gene expression levels of the *n* genes at time points t−m, t−m+1, …, t, and it can be expressed as follows:

(7) $$X^{\left(t\right)}=\left(\begin{array}{cccc} x_{1}^{\left(t-m\right)} & x_{1}^{\left(t-m+1\right)} & \ldots & x_{1}^{\left(t\right)}\\ x_{2}^{\left(t-m\right)} & x_{2}^{\left(t-m+1\right)} & \ldots & x_{2}^{\left(t\right)}\\ \vdots & \vdots & \ddots & \vdots \\ x_{n}^{\left(t-m\right)} & x_{n}^{\left(t-m+1\right)} & \ldots & x_{n}^{\left(t\right)} \end{array}\right)$$

Gene expression at time t+1 can only be calculated from Equation (6) if t≥m. On the contrary, if *t* is less than *m*, the matrix $X^{\left(t\right)}$ is filled with constant initial values obtained by real observations. The practical implementation of the agent function $h_{i}\left(\cdot \right)$ as defined in Equation (6) can be performed using machine learning methods. In fact, they can learn patterns from real observations, which can then be used to predict the gene expression of a gene based on the gene expression of the genes in the previous time steps. For more details on the process of modeling and training an agent, see Section 4.1.2.

### 3.2. Artificial Environmental Setting

Based on the definition of agent in Section 3.1, the gene expression of each gene is regulated by an agent. We use the term *Artificial Environmental Setting (AES)* to refer to a collection of elements, i.e., agents, that interact with each other according to well-defined rules established during the design process. The interactions between the agents are indirect because they do not occur simultaneously, but the inputs of each agent are the product of the other agents from previous time steps. As a result, the process of regulating the gene expression of a gene is more or less influenced by all agents in the AES.

Given an environment with *n* agents, the *state of the environment*, denoted by $x^{\left(t\right)}\in R^{n}$, is defined as a vector of *n* elements containing the gene expression level for each gene at time step *t*. The state of the environment is updated according to Equation (6) computed by each agent. As a result, the state of the environment at time t+1 is defined as:

(8) $$x^{\left(t+1\right)}=\left(x_{1}^{\left(t+1\right)},\;x_{2}^{\left(t+1\right)},\;\ldots ,\;x_{n}^{\left(t+1\right)}\right)=(h_{1}\left(X^{\left(t\right)}\right),\;h_{2}\left(X^{\left(t\right)}\right),\;\ldots ,\;h_{n}\left(X^{\left(t\right)}\right)).$$

Agents in the AES regulate gene expression by taking as input the same matrix $X^{\left(t\right)}$ defined in Equation (7), which is named the *state matrix*. Although each agent has the same state matrix as the input, its agent function $h_{i}\left(\cdot \right)$ is always different as it is modeled to simulate the actual behavior of a gene.

Unlike the state of the environment $x^{\left(t\right)}$, which is a vector containing the gene expression level of all genes and can also be viewed as a collection of the agents’ outputs, the state matrix $X^{\left(t\right)}$ contains the gene expression level of all genes in the previous *m* time steps, and it is used as the agents’ input to calculate the state of the environment at time t+1.

### 3.3. Simulation

We will now study the possibility of simulating gene expression regulation. As previously mentioned, each agent is responsible for regulating the gene expression of a gene. The equation governing the interactions between agents is Equation (8). To simulate gene expression regulation, it is sufficient to begin at a time point (i.e., an initial time point of zero) and proceed to a target time point that we wish to study. To achieve this result, we can use a clock signal that governs the transition from one time step to the next. At each tick of the clock, each agent regulates the gene expression of its gene using Equation (8) and updates the value of the state matrix. This updated value will then be used by agents at the next tick of the clock.

At the start of the simulation (i.e., at t=0), the state matrix has no values yet. As stated in Section 3.1, the state matrix may contain empty values until *t* is less than *m*. Two solutions can be used to address this issue: the first is to set empty values to zero; the second is to populate empty values with real observations from gene expression datasets. In our experiments, we chose the latter solution.

During simulation, it is also possible to manually perturb the environment to observe how regulatory mechanisms respond. This is extremely important in understanding how the gene expression of one gene can affect the regulation of the gene expression in other genes. In this article, when an AES is said to be *perturbed*, we refer to a manual alteration of the state matrix; namely, gene expression levels are updated without using Equation (8).

When there is a change in the state matrix at time *t* due to a perturbation in the gene expression level of the *i*-th gene, it affects the gene expression regulation process of all genes because all agents have the same state matrix as the input. As a result, the state of the environment at time t+1 is always calculated by Equation (8), but taking into account as well the changes in the state matrix made at the previous time.

## 4. Methodology

After having introduced the process of gene regulation modeling using agents and an AES, this section will explore the creation of an AES from a gene expression dataset (Section 4.1) and its application for inferring a gene regulatory network (Section 4.2).

### 4.1. Model Estimation

For simplicity, the process of creating an AES is divided into three phases, labeled *preprocessing*, *learning*, and *evaluation*. The first phase is to normalize the gene expression dataset into a format compatible with our methodology (Section 4.1.1). In the second phase, a metaheuristic algorithm selects the most-appropriate machine learning model for each agent in the AES (one for each gene). The selected model is then trained on a dataset created ad hoc from the original dataset (Section 4.1.2). Once all agents are created, the evaluation process aims to determine the quality of the AES thus created. Ideally, an optimal AES should be able to produce the same response as a real environment, without any external perturbations and with identical initial conditions. In Section 4.1.3, such a process is examined in detail.

#### 4.1.1. Data Preprocessing

Before proceeding with the discussion for creating an AES, it is appropriate to indicate the format of the gene expression dataset we are considering for our experiments. First, there are two versions of the datasets mentioned in Section 2.3: *time-series dataset* and *steady-state dataset*. All experiments reported in this article were performed using the time-series dataset. This is because we want to simulate how gene expression changes over time due to the mutual influence with other genes. As a result, such a result can only be obtained by using a time-series dataset, since it contains the real observations of gene expression over time obtained in the field.

The original datasets were normalized using min–max normalization, which is commonly used for multivariate time-series forecasting [31]. Assuming that the normalized dataset is a matrix $D\in R^{n\times b}$, where *n* is the number of genes and *b* the number of observations for each gene, the dataset used to train each agent must be different. As defined in Equation (6), an agent has as the input the state matrix, which can be represented as a vector of (n·m) elements, and as the output a real value representing the gene expression of the next time point. Consequently, the dataset used to train an agent $A_{i}$ can be represented as $\left(\hat{X},{\hat{Y}}_{i}\right)$, where $\hat{X}$ and ${\hat{Y}}_{i}$ are the input and output sets, respectively. With *m* the number of time steps considered in the state matrix and *b* the number of observations in the dataset *D*, the total number of samples contained in the dataset $\left(\hat{X},{\hat{Y}}_{i}\right)$ is equal to (b−m), since the whole sequence of observations must be split into multiple examples taking into account *m*. In particular, $\hat{X}$ is a matrix of size (b−m)×(n×m) in which each row of the matrix represents a sample and is used as the input to the agent model. The corresponding output is contained in ${\hat{Y}}_{i}$, which is a vector of size (b−m)×1 and contains the observations of the *i*-th gene shifted forward by one time unit.

#### 4.1.2. Learning

The goal of this phase is to create a collection of agents, each of which forecasts the expression of a gene over time. Each agent is created independently of the others and can have a different architecture. This is because an agent must learn how to regulate the gene expression of a single gene. The procedure for creating agents from gene expression data is the same for all agents. For simplicity, we assume that we are creating an agent that refers to the *i*-th gene, and such an agent will be referred to below as $A_{i}$. Each agent is associated with a different dataset $\left(\hat{X},{\hat{Y}}_{i}\right)$, which is split into two parts to define a training set and a test set on which the agent is trained and evaluated. To create an agent, an appropriate machine learning method is selected that can better generalize on this dataset than others. This is because each machine learning method has its own properties and characteristics, so different methods may perform differently on the same dataset. Indeed, we have already proven in [32,33] that different machine learning models together can increase overall accuracy in multivariate time-series forecasting. In our experiments, we considered three different types of neural networks used to model the agent function $h_{i}\left(\cdot \right)$. Table 2 lists the neural networks considered and their basic architecture, as well as the layers used for each neural network. A distinctive feature of our methodology is that the hyperparameters of each neural network listed are not fixed, but are selected during the hyperparameter optimization procedure by a metaheuristic algorithm [34]. More details can be found in Appendix A.

One approach for selecting the optimal neural network architecture for an agent is to train all three available types of neural networks, namely *Fully Connected Neural Network* (FCNN), *Convolutional Neural Network* (CNN), and *Recurrent Neural Network* (RNN), on the training set and evaluate their performance on the test set. The neural network with the lowest error is then chosen as the agent $A_{i}$ [35]. However, this approach may not be the most-effective way to select the agent architecture in our model, as the final performance depends not only on a single agent, but on a group of agents that interact with each other through the state matrix. Additionally, this approach can be computationally expensive. More details about the algorithm used to select agent architectures can be found in Section 4.1.4.

#### 4.1.3. Evaluation

We used different evaluation metrics to assess the accuracy of gene regulation. Typically, the gene expression dataset that is being analyzed contains multiple measurements of expression levels over time, referred to as *experiments*. However, when selecting the training and testing datasets, not all *k* experiments are considered. Instead, a smaller subset is reserved for evaluating the performance of the AES. To evaluate the performance of the AES, we utilize a dataset $D_{Y}\in R^{n\times b}$. This dataset consists of *b* observations and *n* genes, with each element representing the average of *k* experiments. These experiments include both those used to create the agents, as well as those that were not used. We used the average number of experiments *k* to validate our model because it provides a more-accurate representation of the underlying biological system. A single experiment may not capture the full range of biological variability and may be subject to measurement noise or other experimental artifacts. By averaging multiple experiments, we can reduce the impact of these factors and obtain a more-reliable estimate of the true biological behavior. Furthermore, using a single experiment not used in training may not be sufficient to fully evaluate the performance of the AES since it may exhibit different behaviors or responses under different experimental conditions. By utilizing multiple experiments, we can capture a broader range of biological variability and better assess the robustness and scalability of the AES.

Predicted gene expression is determined by the environment simulation, as described in Section 3.3. In this case, the initial values of the AES are taken from the first *m* observations of the target dataset $D_{Y}$, and the simulation time is equal to the number of observations contained in $D_{Y}$. During the simulation, the state of the environment changes at each time point and its values are recorded in a matrix $D_{X}\in R^{n\times b}$ and called *predicted gene expression*. Given the target gene expression $D_{Y}$ and the predicted gene expression $D_{X}$, it is possible to use similarity metrics to quantify the accuracy of the AES with real numbers. Thus, such values, which we call *AES reliability* (*R*), are a measure of how much our AES is able to replicate the real behavior. Formally, it is defined as:

(9) $$R_{metric}\left(D_{X},D_{Y}\right)=\frac{1}{n}\sum_{i=1}^{n}\left|metric\left(d_{x_{i}},d_{y_{i}}\right)\right|,$$

where *n* is the number of genes; $d_{x_{i}}$ and $d_{y_{i}}$ are two vectors of (b−m) elements, containing, respectively, the predicted gene expression values and the target gene expression values over time of the *i*-th gene. To evaluate the accuracy of the predictions made by each agent on the AES, we only use (b−m) out of *b* observations in total. This is because the first *m* elements predicted from the AES are identical to those of the target dataset, since they share the same initial conditions. Therefore, we excluded these elements and took into account only actual predictions. In addition, the function denoted by $metric\left(d_{x_{i}},d_{y_{i}}\right)$ is a metric function that returns a real value obtained by comparing these two vectors. Two metrics were used to compute the AES reliability, namely *Pearson correlation* (ρ) and *cosine similarity* (η) [36], whose mathematical expressions are

(10) $$\rho \left(d_{x_{i}},d_{y_{i}}\right)=\frac{\sum_{j=1}^{\left(b-m\right)}\left(d_{y_{ij}}-\bar{d_{y_{i}}}\right)\left(d_{x_{ij}}-\bar{d_{x_{i}}}\right)}{\sqrt{\sum_{j=1}^{\left(b-m\right)}{\left(d_{y_{ij}}-\bar{d_{y_{i}}}\right)}^{2}}\sqrt{\sum_{j=1}^{\left(b-m\right)}{\left(d_{x_{ij}}-\bar{d_{x_{i}}}\right)}^{2}}},$$

(11) $$\eta \left(d_{x_{i}},d_{y_{i}}\right)=\frac{\sum_{j=1}^{\left(b-m\right)}d_{y_{ij}}d_{x_{ij}}}{\sqrt{\sum_{j=1}^{\left(b-m\right)}d_{y_{ij}}^{2}}\sqrt{\sum_{j=1}^{\left(b-m\right)}d_{x_{ij}}^{2}}},$$

where $\bar{d_{y_{i}}}$ and $\bar{d_{x_{i}}}$ represent, respectively, the mean of the target and the predicted observations of the *i*-th gene. These two similarity metrics are commonly used to quantify, with a real value, whether the predicted time-series is as similar as possible to the target time-series [36]. Below, we refer to these two versions of environment reliability as $R_{\rho }$ and $R_{\eta }$ for reliability based on the Pearson correlation and cosine similarity, respectively.

In addition to reliability, we used two other metrics to quantify the error between gene regulation by the AES and actual behavior. We define the *AES error* (Err) as the average error of the single-gene regulations, that is:

(12) $$Err_{metric}\left(D_{X},D_{Y}\right)=\frac{1}{n}\sum_{i=1}^{n}error\left(d_{x_{i}},d_{y_{i}}\right).$$

The error metrics we have used are the *Mean-Squared Error* (MSE) and the *Mean Absolute Error* (MAE), defined as follows:

(13) $$MSE\left(d_{x_{i}},d_{y_{i}}\right)=\frac{1}{b-m}\sum_{j=1}^{\left(b-m\right)}{\left(d_{y_{ij}}-d_{x_{ij}}\right)}^{2},$$

(14) $$MAE\left(d_{x_{i}},d_{y_{i}}\right)=\frac{1}{b-m}\sum_{j=1}^{\left(b-m\right)}\left|d_{y_{ij}}-d_{x_{ij}}\right|.$$

We will refer to these two versions of AES reliability as $Err_{MSE}$ and $Err_{MAE}$ for reliability based on the MSE and MAE, respectively.

#### 4.1.4. Optimization Algorithm

The selection of the agent architecture type is crucial for improving the accuracy of gene expression regulation, as previously mentioned. To identify the optimal neural network architecture for an agent among those listed in Table 2, we used a genetic algorithm for integer programming [37]. This algorithm assigns an integer value to each neural network type, and so, it allows us to determine the most-effective combinations of agents. By employing this algorithm, we can select the best-possible agents to enhance the accuracy of gene expression regulation. A solution of the genetic algorithm is a vector of size equal to the number of agents present in the AES and so the number of genes *n*. Each element of the solution vector defines the type of architecture that is assigned for the corresponding agent, which in our case can be just one of the three types, FCNN, CNN, and RNN.

The metaheuristic search generates good solutions according to the genetic operators mutation and crossover and discards all solutions with a low fitness value. The fitness of a solution is a real value that reflects the quality of that solution: the higher the fitness value, the higher the quality of the solution is. Algorithm 1 shows the pseudo-code used to compute the fitness of a solution $S\in {\left\{FCNN,CNN,RNN\right\}}^{n}$.
**Algorithm 1:** Pseudo-code to compute the fitness of a possible configuration of an environment.
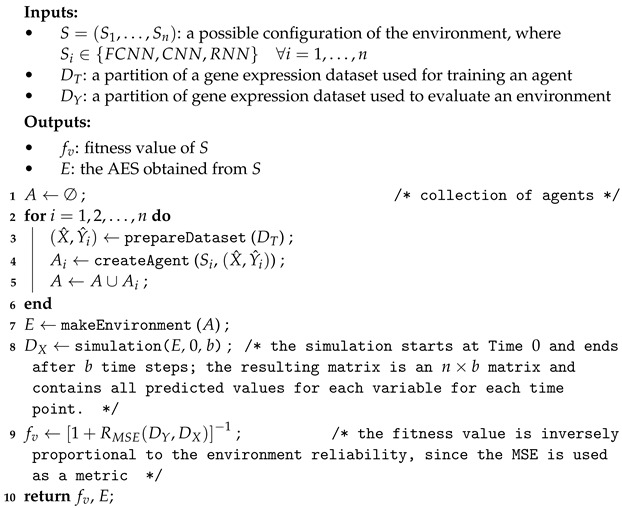


For each element of the solution, the corresponding agent is created. To explain the process of creating an agent, consider the following example. We assume that $S_{i}\in S$ refers to an architecture of type RNN. The first step is to prepare the dataset $\left(\hat{X},{\hat{Y}}_{i}\right)$ that will be used to train a recurrent neural network, as indicated in Section 4.1.1 (Line 3). The procedure labeled createAgent in Line 4 has the function of creating the *i*-th agent based on the recurrent neural network and training it with the previously created dataset. For this procedure, we used a hyperparameter-optimization algorithm in order to find the best hyperparameters for the layers of the recurrent neural network. The hyperparameter-optimization algorithm tests different combinations of hyperparameters for the considered neural network architecture on the dataset $\left(\hat{X},{\hat{Y}}_{i}\right)$. Finally, the recurrent neural network with the least error is returned and assigned as the *i*-th agent of the environment (Line 5). Appendix A provides additional details on the process of selecting hyperparameters for the neural network architectures.

Once all agents have been created according to *S*, the resulting environmental setting is evaluated using the methods described in Section 4.1.3 (specifically, Lines 8 and 9). To determine the fitness of *S*, we use the mean-squared error (as defined in Equation (13)) as a metric function in Equation (9). Other than the fitness function, the other operators of the discrete genetic algorithm are standard. In our experiment, we used a population size of 10, a mutation probability of 0.2, a crossover probability of 0.8, and tournament selection with a tournament size of 3. We also set the maximum number of evaluations to 100.

### 4.2. Inferring a Gene Regulatory Network

We will now describe a methodology that allows us to extract a gene regulatory network from simulations conducted in our AES. First, we simulate the gene expression until all expression levels are within a certain range, then we employ some perturbations to alter the gene expression of one gene and then measure the resulting changes in the expression of other genes. By analyzing these changes, we can infer the regulatory relationships between genes and construct a gene regulatory network. Finally, we use metrics to evaluate the resultant gene regulatory network.

#### 4.2.1. Perturb the State Matrix

To better understand this procedure, let us consider an example. Suppose we want to test whether the *i*-th gene regulates the expression of other genes. At time *t*, we introduce a perturbation of the *i*-th agent to manually alter its gene expression regulation. A perturbation can be defined as a mathematical function $\varphi_{i}:\left[t,t+\varphi_{d}\right]\subset N\to \left[0,G_{i_{\max}}\right]\subset R$, where *t* is the time expressed in time steps in which the *perturbation function* operates and $\varphi_{d}$ is the duration of the perturbation. A perturbation function on gene $G_{i}$ returns a real value between $\left[0,G_{i_{\max}}\right]$ for each time step that represents the expression level of gene $G_{i}$. This value is independent of the expression levels of other genes, and it is used to update the state matrix that contains the expression levels of all genes at each time step. We denote by $G_{i_{\max}}$ the maximum expression level observed for gene $G_{i}$ in the dataset.

The application of the perturbation function $\varphi_{i}\left(\cdot \right)$ to the *i*-th gene affects the state matrix. In particular, the elements of the state matrix along the *i*-th row are not computed by the agent function as in the case without perturbation (Equation (6)), but their values depend directly on the perturbation function. Assuming that $1<\varphi_{d}<m$, the state matrix of the environment at time t+m can be expressed as follows:

(15) $$X^{\left(t+m\right)}=\left(\begin{array}{ccccccc} x_{1}^{\left(t-1\right)} & x_{1}^{\left(t\right)} & x_{1}^{\left(t+1\right)} & \ldots & x_{1}^{\left(t+\varphi_{d}\right)} & \ldots & x_{1}^{\left(t+m\right)}\\ \vdots & \vdots & \vdots & \ddots & \vdots & \ddots & \vdots \\ x_{i}^{\left(t-1\right)} & \varphi_{i}\left(t\right) & \varphi_{i}\left(t+1\right) & \ldots & \varphi_{i}\left(t+\varphi_{d}\right) & \ldots & x_{i}^{\left(t+m\right)}\\ \vdots & \vdots & \vdots & \ddots & \vdots & \ddots & \vdots \\ x_{n}^{\left(t-1\right)} & x_{n}^{\left(t\right)} & x_{n}^{\left(t+1\right)} & \ldots & x_{n}^{\left(t+\varphi_{d}\right)} & \ldots & x_{n}^{\left(t+m\right)}. \end{array}\right)$$

**Figure 4:**
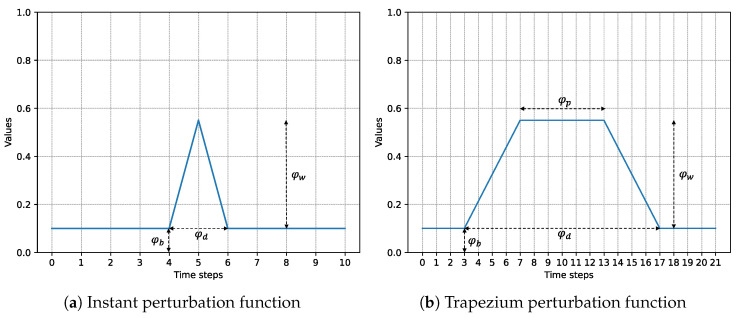
Representation of the perturbation functions considered. The two perturbation functions (**a**,**b**) share the same parameters $\varphi_{b}$, $\varphi_{d}$, and $\varphi_{w}$, which denote the initial value of the *i*-th gene to be perturbed, the overall duration of the perturbation and the width of the perturbation, respectively. Additionally, the trapezium perturbation function (**b**) requires another parameter $\varphi_{p}$, which represents the number of time steps for which the peak value is maintained.

Depending on the type of perturbation function selected, different behaviors can be observed. In our experiments, we tested several types of perturbation functions. However, we only report two types of perturbation functions that have achieved the best performance in terms of accuracy in identifying gene regulators. These two types are depicted in Figure 4. In our previous work [6], the approach that we used to perturb the regulation of a gene by an agent can be approximated in a similar way by using the function shown in Figure 4a, called the *instant perturbation function*. This function increases immediately the gene expression of the perturbed gene at time *t* by a quantity indicated by $\varphi_{w}$. An improvement of the instant perturbation function is the *trapezium perturbation function* (Figure 4b), which consists of increasing gradually the value of gene expression of a gene and maintaining the peak value for a certain number of time steps indicated by $\varphi_{p}$, then decreasing gradually the gene expression value of the perturbed gene until its initial value $\varphi_{b}$ is reached. With this behavior, it is possible to observe how other genes are influenced throughout the duration of the perturbation and measure consequently the eventual dependence among genes.

#### 4.2.2. Regulatory Value and Regulatory Matrix

To determine which genes are regulated by the *i*-th gene after a perturbation affects its regulation process, it is necessary to identify the genes whose expression levels are significantly changed by that perturbation. Therefore, if a group of genes shows a noticeable change in their expression levels, then gene $G_{i}$ may be their regulator. The procedure to establish the genes regulated by gene $G_{i}$ consists of the following steps:

1. The simulation starts with initial conditions and runs until all the variables reach a steady state within a certain range. The time point at the end of this step is $t_{f}$;
2. The simulation continues for *m* more time steps to ensure the stability of the environment. The time point at the end of this step is $t_{f}+m$;
3. A perturbation function $\varphi_{i}\left(\cdot \right)$ is applied to gene $G_{i}$ for $\varphi_{d}$ time steps. The time point at the end of this step is $t_{f}+m+\varphi_{d}$. The perturbation duration is a hyperparameter, and we set it to *m*;
4. The simulation runs for *m* more time steps until the state matrix stabilizes after the perturbation. The time point at the end of this step is $t_{f}+\Delta t_{r}$, where $\Delta t_{r}$ is $\left(2m+\varphi_{d}\right)$ and represents the *instability interval* caused by the perturbation;
5. Changes in the gene expression level of all genes between $t_{f}$ and $t_{f}+\Delta t_{r}$ are recorded in a matrix $X_{r}\in R^{\Delta t_{r}\times n}$;
6. For each gene, the *regulatory value* is computed as the slope in radians of a linear regression model fit on the values between $t_{f}$ and $t_{f}+\Delta t_{r}$.

This procedure estimates the regulatory value of each gene in the environment with respect to another gene. We denote by $r_{ij}$ the regulatory value of gene $G_{i}$ on gene $G_{j}$, which quantifies the impact of a perturbation of gene $G_{i}$ on the regulation of gene $G_{j}$. This allows us to assess the degree of association between these two genes. Essentially, if we extract the *j*-th column of the matrix $X_{r}$ and denote it by $X_{r_{j}}\in R^{\Delta t_{r}}$, the regulatory value is computed as:

(16) $$r_{ij}=\arctan\left(\alpha_{j}\right),$$

where $\alpha_{j}$ is the slope of the linear regression fit on the values contained in $X_{r_{j}}$. Therefore, the regulatory value is always a quantity defined within the interval $\left[-\frac{\pi }{2},\frac{\pi }{2}\right]$ according to the definition of the arctangent.

In our previous approach [6], we computed the regulatory value by comparing the state of the environment at time $t_{f}+\Delta t_{r}$ with the one at time $t_{f}$. This allowed us to determine whether a perturbation of the *i*-th gene affected the regulation of the gene expression of some genes. However, we realized that this approach did not take into account all the intermediate states assumed during the perturbation. By using this new version, instead, we consider the influence that the perturbed gene $G_{i}$ has on the other genes and avoid the particular scenario mentioned above. The gene regulatory network can be inferred from the regulatory genes obtained from this information. To figure out which gene are regulatory genes, we introduce the concept of a *regulatory matrix*, denoted by $R\in R^{n\times n}$, which is a square matrix that contains the regulatory values for each pair of genes. The regulatory values are computed for each gene in response to a perturbation of another gene $G_{i}$, where i=1,2,…,n.

#### 4.2.3. Interaction Probability Matrix

After having computed the regulatory matrix as above described, we must determine the gene regulatory network. A regulatory value between a pair of genes $\left(G_{i},G_{j}\right)$ is defined as a measure of how gene $G_{j}$ is affected by gene $G_{i}$. The higher this value in terms of absolute value, the higher the probability that gene $G_{i}$ is a regulatory gene of gene $G_{j}$. This section describes a procedure used to transform the regulatory matrix obtained by perturbing each gene of the AES into a probability matrix. The probability matrix is denoted by $P\in R^{n\times n}$, where the generic element $p_{ij}$ represents the probability that the *i*-th gene is regulated by the *j*-th gene. This value is computed directly by the respective regulatory value $r_{ij}$ in the regulatory matrix.

A gene $G_{j}$ can be considered to be regulated by another gene $G_{i}$ (*i* and *j* might be the same) only if the influence of the gene expression of gene $G_{j}$ due to a perturbation of gene $G_{i}$ exceeds a certain activation threshold. On the other hand, $G_{j}$ is inhibited by $G_{i}$ if the corresponding regulatory value is less than an inhibition threshold. Therefore, given the activation threshold and the inhibition threshold for each gene, denoted, respectively, by the symbols $\tau_{j}^{p}$ and $\tau_{j}^{n}$, the regulatory value $r_{ij}$ between genes $G_{i}$ and $G_{j}$, and also taking into account that $r_{ij}$ is bounded between $\left[-\frac{\pi }{2},\frac{\pi }{2}\right]$, the probability that $G_{i}$ is a regulatory gene of gene $G_{j}$ is given by the following formula:

(17) $$p_{ij}=\left\{\begin{array}{c} \frac{1}{2}+\frac{1}{2}\frac{r_{ij}-\tau_{j}^{n}}{R_{j_{\min}}-\tau_{j}^{n}}\;\mathrm{if}\;R_{j_{\min}}\leq r_{ij}\leq \tau_{j}^{n},\;\\ -\frac{r_{ij}^{2}}{2\tau_{j}^{p}\tau_{j}^{n}}+\frac{\tau_{j}^{p}+\tau_{j}^{n}}{2\tau_{j}^{p}\tau_{j}^{n}}r_{ij}\;\mathrm{if}\;\tau_{j}^{n}<r_{ij}<\tau_{j}^{p},\;\\ \frac{1}{2}+\frac{1}{2}\frac{r_{ij}-\tau_{j}^{p}}{R_{j_{\max}}-\tau_{j}^{p}}\;\mathrm{if}\;\tau_{j}^{p}\leq r_{ij}\leq R_{j_{\max}}, \end{array}\right.$$

where $R_{j_{\max}}$ and $R_{j_{\min}}$ are, respectively, the maximum and minimum value.

**Figure 5:**
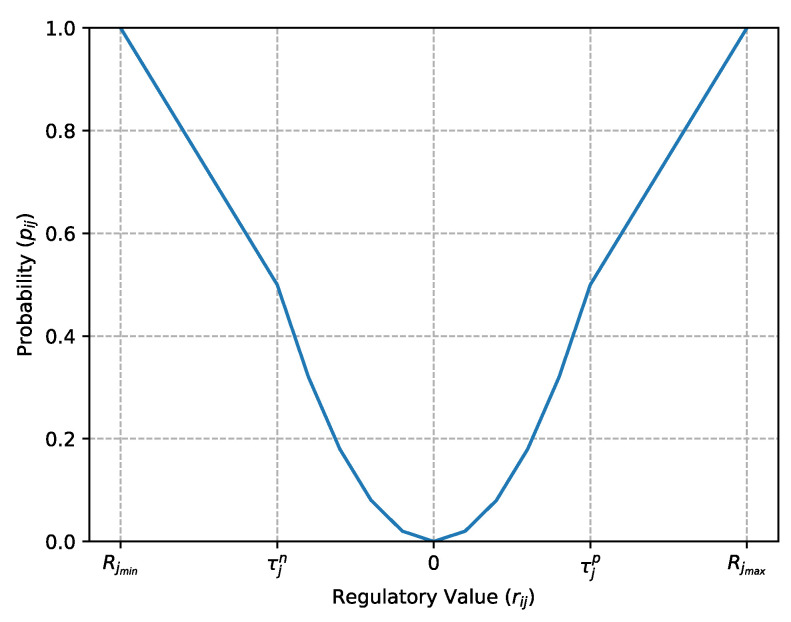
Function to transform a regulatory value into a probability value.

Figure 5 displays how a regulatory value $r_{ij}$ is transformed into a probability value $p_{ij}$.

To compute the probability matrix *P* and, thus, to determine if $G_{i}$ is a regulatory gene of $G_{j}$, the activation and inhibition thresholds need to be determined for each gene. The performance of our methodology depends on the criteria used for choosing the threshold value. The threshold values of the *j*-th gene are computed taking into account how this gene responds to the perturbations on all the other genes, which can be observed by looking at the regulatory values presented in the *j*-th column of the regulatory matrix. The activation threshold of gene $G_{j}$ denoted by $\tau_{j}^{p}$ is the median of the positive elements across the *j*-th column of the regulatory metric *R*:

(18) $$\tau_{j}^{p}=med\left(\left\{r_{kj}\;|\;r_{kj}\in R\;and\;r_{kj}>0\right\}\right).$$

In a dual manner, the inhibition threshold of gene $G_{j}$ denoted by $\tau_{j}^{n}$ is the median of the negative elements across the *j*-th column of the regulatory metric:

(19) $$\tau_{j}^{n}=med\left(\left\{r_{kj}\;|\;r_{kj}\in R\;and\;r_{kj}<0\right\}\right).$$

Choosing the right threshold value is important because it directly affects the accuracy and reliability of the inferred network. On the one hand, if the threshold values are too high, many true interactions may be missed, resulting in a sparse network that does not accurately reflect the underlying biology. On the other hand, if the threshold values are too low, many false interactions may be included, resulting in a dense network that contains many spurious connections. Therefore, it is important to choose threshold values that balance the trade-off between sensitivity (the ability to detect true interactions) and specificity (the ability to exclude false interactions). This can be achieved by carefully considering the distribution of regulatory values and the underlying network structure and by validating the inferred network using independent experimental data. At this initial stage, we have chosen to set the threshold value at the median of the regulatory values based on empirical testing. Indeed, biological networks are typically not highly dense, and therefore, the use of the median to compute threshold values is a reasonable approach for this type of network. However, in rare cases where the regulatory network is highly connected with high density, this strategy may need to be revised, but to our knowledge, this is a theoretical case that is not realizable in an actual biological network.

#### 4.2.4. Evaluation of a Gene Regulatory Network

After inferring a gene regulatory network, it is common to use performance metrics such as the accuracy, precision, specificity, and sensitivity to evaluate the quality of the predicted gene regulatory network. However, in cases where the output of the methodology is a probability matrix *P* that contains the probability that a gene is a regulatory gene of another gene, the Area Under the Curve of the Receiver Operating Characteristic (AUC-ROC) is used as a performance metric instead [38]. Given a probability matrix *P* obtained from the regulatory matrix *R*, it is possible to define a probabilistic network in which each arc of such a network has a probability of appearing or not. Therefore, a vector $\hat{p}\in R^{n^{2}}$ can be defined that contains the probabilities of all possible arcs between genes in the network. On the other hand, we define $p\in {\left\{0,1\right\}}^{n^{2}}$ as a binary vector that represents the presence (1) or absence (0) of an arc in the target network. Knowing $\hat{p}$ and p makes it possible to compute the AUC metric to determine the quality of the prediction.

## 5. Results

To show the benefits of our methodology, we present the results that we obtained from our experiments in this section. The discussion is structured as follows:

- We start by proving that our method of creating models, capable of predicting gene expression levels over time, works well on all datasets, including both real and artificial datasets (Section 5.1).
- Then, we show that our strategy of using metaheuristics to select the appropriate neural network architecture for each agent is the key to accurate simulation (Section 5.2).
- Subsequently, we aim to prove that our AES responds and regulates the gene expression in accordance with the behavior present in the real world under specific perturbations of specific genes (Section 5.3).
- Finally, we present and discuss the results obtained in terms of inferring a gene regulatory network, comparing our results with the state-of-the-art methodology (Section 5.4).

### 5.1. Environment Reliability Analysis

The first step in validating our methodology, as described in previous sections, is to demonstrate the ability of our AES to learn how to regulate gene expression from time-series datasets. As previously mentioned, each agent must learn gene expression regulation that is as similar as possible to the real gene expression regulation process. Therefore, we conducted experiments with the datasets described in Section 2.3 to demonstrate how our model can simulate the regulation of gene expression for a group of genes. In these experiments, we used both real datasets obtained by actual observations of gene expression and the artificial datasets listed in Table 1.

Table 3 provides a concise summary of the results obtained from all the datasets used in the study. Each row in the table corresponds to a specific dataset containing a particular number of genes. To obtain these results, we first preprocess the dataset according to the guidelines outlined in Section 2. Subsequently, we create an AES using the method specified in Section 4.1.2. Finally, we evaluate the performance of the model in this environment according to the criteria described in Section 4.1.3. The environment reliability and environment error defined, respectively, in Equations (9) and (12) are reported in the table. Note that the reliability values are always between 0 and 1, while the environment error has no upper bound and should ideally be as small as possible. In the table below, for convenience, we also report the reliability measurements in percentage terms.

All results and plots presented in this section were obtained using the methodology previously described. The number of time steps was set to *m*, and the size of the state matrix was set to 10. Since each dataset has a different number of experiments, we applied the following rule to select which experiments to use to create the AES and, in turn, which experiments to use to evaluate it: for each dataset, 60% of the available experiments were used to create the AES, while an experiment obtained by averaging all experiments was used to evaluate it. These parameters were used for all experiments. As mentioned earlier, to simulate regulation, it is necessary to set the initial state matrix to correspond to the first *m* observations of the dataset used for environment evaluation.

**Figure 6:**
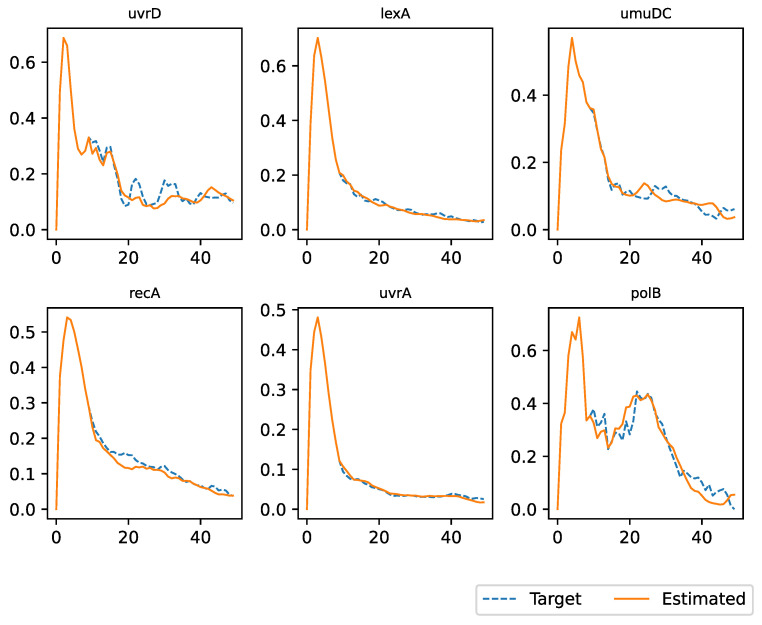
Regulation of expression of eight genes using the dataset with ID 17.

**Figure 7:**
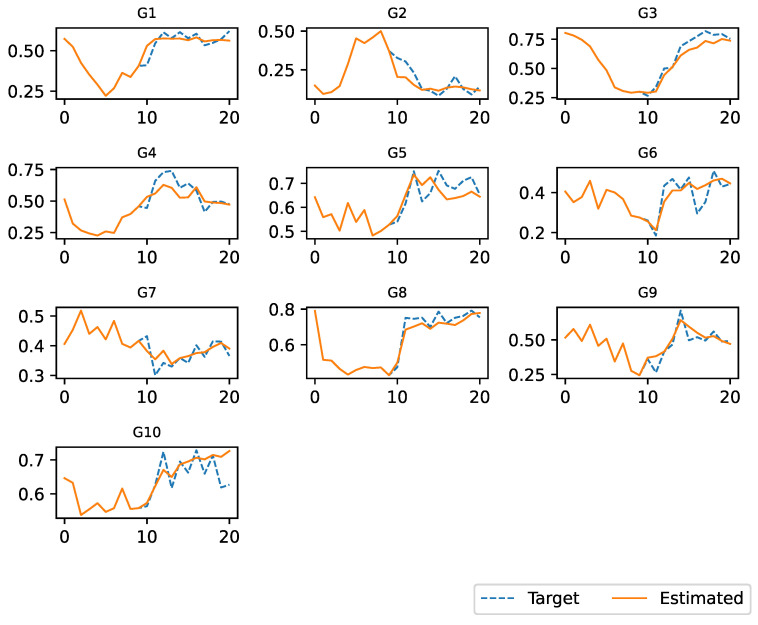
Regulation of expression of ten genes using the dataset with ID 10.

Figure 6 and Figure 7 show a visual comparison between the regulation of gene expression by the AES and that observed in the field. The first 10 steps correspond to those of the target dataset. After 10 steps, the expression for each gene was estimated by the AES. The simulation ended when the number of steps was equal to the number of observations contained in the dataset.

The two figures refer to two datasets with IDs equal to 17 and 10, respectively. We have chosen to report only on these two datasets because they contain a small number of genes and are easier to display. However, the same considerations can be applied to the other datasets. In these figures, the blue line represents the real gene expression data, while the orange lines represent the regulation of gene expression estimated by an AES. The horizontal axis represents relative time and not absolute time, while the vertical axis represents gene expression normalized in an interval between 0 and 1.

### 5.2. Optimization Analysis

This subsection highlights the advantages of using optimization algorithms to create an AES with the highest reliability, according to the definition of environment reliability given in Equation (9). In order to generate a group of agents that regulate gene expression, we employ a two-level optimization algorithm. The first level determines the type of neural network that models the agent’s function, while the second level identifies the optimal hyperparameters for that neural network to minimize the error. We evaluated various configurations of the AES on the SOS DNA Repair dataset, which involves repairing DNA damage caused by environmental stresses. The results of our optimization technique (outlined in Section 4.1.4) are presented in Table 4. The table compares the performance of optimized and non-optimized AES. The agent type column indicates the neural network architecture used for all agents (FCNN, CNN, or RNN). The hyperparameter optimization column shows whether the hyperparameters of a neural network were tuned or not. The remaining columns are the metrics used for the evaluation. The last row shows the results of our optimized version (also reported in Table 3). The results are compared to a baseline without optimization and a partial optimization, where all agents have the same neural network architecture (FCNN, CNN, or RNN), while the partial optimization only tunes the hyperparameters of a neural network. Our optimization method outperforms both alternatives.

The results reported in Table 4 suggest that gene expression regulation can vary dynamically over time and that some models are more suitable than others for capturing this variation.

**Figure 8:**
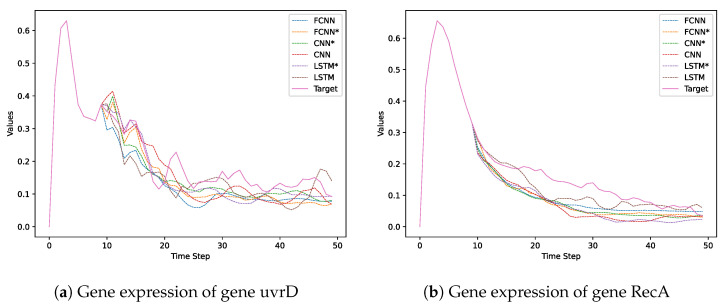
Comparing gene expression regulation performed by different models on the SOS DNA Repair dataset. The solid lines represent the actual values of gene expression for the two selected genes, while the dashed lines are the predictions made by the models.

To illustrate this point, Figure 8 compares how different types of models regulate the expression levels of two out of eight genes in the SOS DNA Repair dataset. We selected these two genes because they show a clear difference between models. We marked with an asterisk (*) all the neural networks that have been optimized according to Section 4.1.4, while the others have not been optimized. We also included the actual values of gene expression for these two genes. Finally, in Table 5 are reported the neural network architecture assigned to each agent/gene in order to maximize the AES reliability using SOS DNA Repair dataset.

### 5.3. Inferring Method Validation

**Figure 9:**
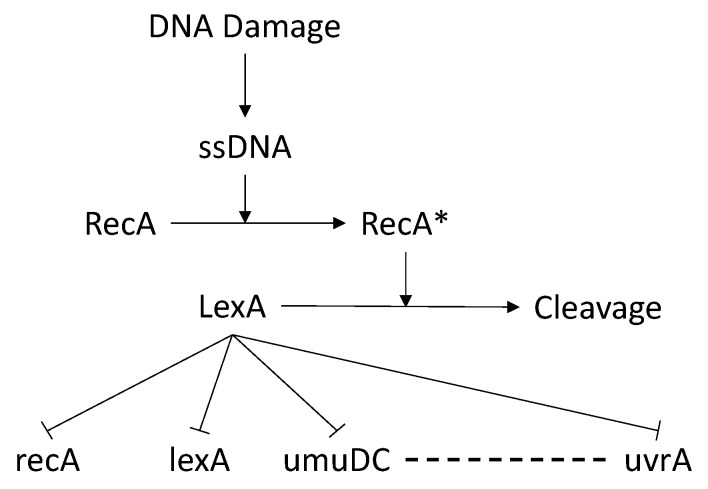
SOS DNA Repair [39].

In this section, we present a case study on the SOS DNA Repair dataset to illustrate the robustness of the proposed method. We chose this dataset because it reflects real observations of genes involved in the biological process of DNA repair. Figure 9 shows the GRN target that represents the gene interactions.

**Figure 10:**
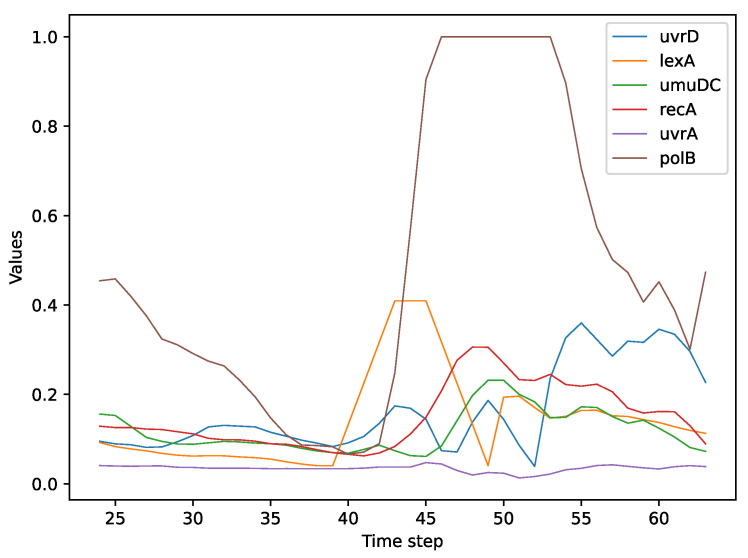
Trapezium perturbation function on the gene lexA in the SOS DNA Repair dataset.

One of the key genes in this process is lexA, which has a strong influence on and, in turn, is influenced by other genes. lexA is a transcriptional repressor that regulates the expression of genes involved in DNA damage repair and population dynamics in Escherichia coli. The SOS response is an inducible pathway that allows bacteria to cope with various types of DNA lesions by activating DNA repair mechanisms and increasing mutation rates [40]. When DNA damage occurs, the recA protein becomes activated by binding to single-stranded DNA and stimulates the autocatalytic cleavage of lexA, resulting in derepression of the SOS genes [41]. lexA also regulates its own expression and that of recA. To examine whether this behavior can be replicated by using our perturbation method, we applied a trapezoid perturbation function on the gene lexA after the environment had reached stability. As described in Section 4.2.1, if a perturbation of a gene affects the regulation of the gene expression of other genes, then there is a relation between them. Figure 10 shows how the perturbation of lexA influences the expression of all other genes. This plot confirms two important principles that were stated above: (1) a change in the expression level of lexA induces a significant variation in the expression of all the other genes involved in the SOS DNA repair process; and (2) lexA regulates the gene expression of recA.

Table 6 shows the regulatory matrix extracted from the perturbations of all genes according to Equation (16). The second row indicates the regulatory values obtained from the perturbation of lexA: in green are reported the relationship correctly inferred, while in red the wrong ones. As seen in Section 4.2.1, the columns represent the regulatory value of the corresponding gene measured during a perturbation of the related gene in the corresponding table row. To confirm the presence or absence of relationships, we report the absolute values. The greater the value, the higher the probability of an interaction between two genes. As depicted in Figure 9, perturbing the gene lexA has a significant impact on the genes uvrD, umuDC, recA, and polB. Conversely, perturbations of other genes do not affect these genes as much as lexA, as there are no arcs between those genes and lexA, except for recA. When analyzing the effect of perturbing recA on lexA, we observe a value of approximately $4.77\times 10^{-2}$, confirming recA as a regulator of lexA (green cells). Although polB has a value of $6.4840\times 10^{-2}$, which is close to recA, it is not considered a regulatory gene of lexA, to the best of our knowledge, and in this case, we have a false positive (red cells). On the other hand, a perturbation of lexA does not significantly affect the gene uvrA, as the regulatory value obtained is very small. However, this contradicts the actual relationship between lexA and uvrA, where lexA regulates uvrA as well. This behavior represents a false negative for our methodology.

It is important to note that these values may not reflect the real behavior for several reasons. The first reason is that we trained the AES using limited observations. Moreover, these values need to be validated in the field by using microarray technologies. However, with this method, we can only assert that there exists a relationship between lexA and all the genes that are most affected by the perturbation. This behavior can be observed in Figure 9.

### 5.4. Gene Regulatory Networks

**Figure 11:**
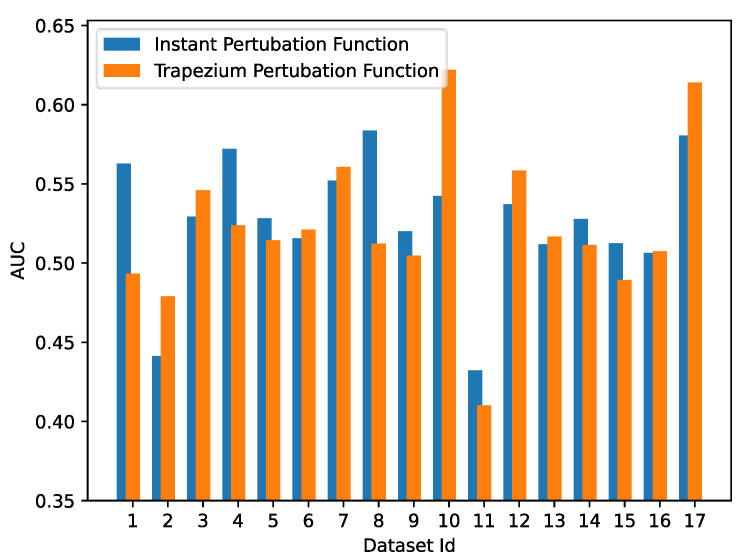
Results obtained by our methodology taking into account the two types of perturbations: instant perturbation function and trapezium perturbation function. The dataset ID is an identifier that represents the dataset used in that experiment. The full list of datasets is reported in Table 3.

We now discuss the inferring of a gene regulatory network given an AES trained by gene expression datasets. We used the AUC-ROC as a metric to evaluate the quality of our predictions. The results are reported in Figure 11. We inferred a gene regulatory network considering two distinct perturbation functions, namely the instant and trapezium perturbation function (see Section 4.2.1) and the same datasets used in Table 3. To perform these experiments, we chose *m* as the perturbation duration $\varphi_{d}$ and half of the maximum value of gene expression as the perturbation width $\varphi_{w}$. In the trapezium perturbation function, we chose a $\varphi_{p}$ equal to 2.

The study found that gene regulatory networks inferred using an instant perturbation function are more accurate when using artificial datasets than real datasets. Conversely, gene regulatory networks inferred using a trapezium perturbation function achieve better performance than those using an instant perturbation function when using real datasets. Indeed, artificial datasets are typically generated using a random distribution as a baseline, which can induce a rapid change in gene expression immediately. This is the reason why, with artificial datasets, an instant perturbation function works better. On the other hand, in an AES modeled by actual observations such as the *SOS DNA Repair dataset* (Dataset ID equal to 17), agents’ reactions occur after several time steps. As a result, our approach seems to be more suitable for use with real datasets, as the agents are able to learn the actual regulation of gene expression. In an artificial dataset, the gene expressions at initial time (t=0) assume random values different from zero. This behavior is not present if we consider actual observations, such as SOS DNA Repair, where the gene expression at the initial time is set to zero because the system is in a quiet state.

**Figure 12:**
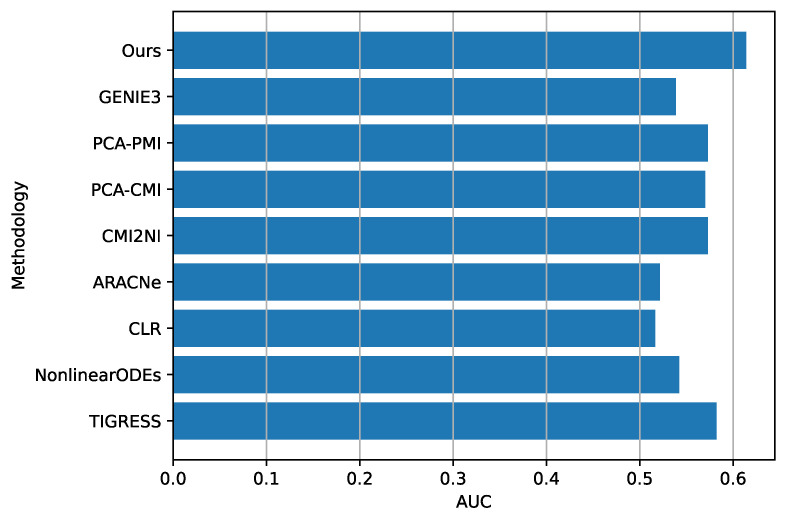
This figure presents a comparison between our approach (labeled as “Our”) and the state-of-the-art method for the SOS DNA Repair dataset, with the results sourced from [11].

**Figure 13:**
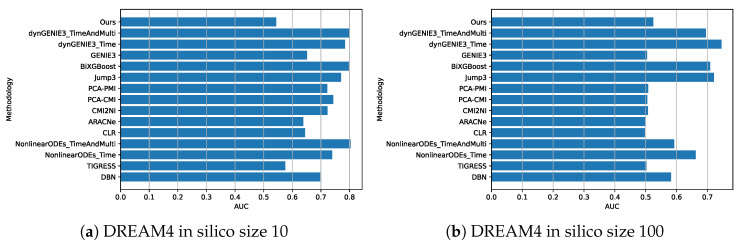
This figure compares the performance of our approach (labeled as “Our”) with the state-of-the-art method for DREAM4 datasets. The values represent the average of the Area Under the Curve (AUC) obtained for each instance, as per [11], where the results from other methods are used for comparison.

If we consider the AUC value obtained with SOS DNA Repair, which was recorded as 0.61389, upon comparison with the state-of-the-art, we observe that it surpasses the current best result as stated in [11]. To provide a clear comparison for the reader, we have included a figure (Figure 12) that displays the AUC values achieved by other inference methods, as per the experiments conducted in the aforementioned study. Conversely, when it comes to artificial datasets, such as those from the DREAM4 Challenges with 10 and 100 genes (dataset IDs 7 to 16), our results, when compared to state-of-the-art methods, are not optimal, as can be seen in Figure 13. However, it is worth noting that our model appears to perform slightly better with 100 genes (Figure 13b) than with datasets containing only 10 genes (Figure 13a).

## 6. Conclusions and Future Work

Gene expression is a fundamental process in living organisms that involves the synthesis of gene products such as proteins using genetic information. It plays a crucial role in many biological processes such as development, differentiation, and disease. Gene Regulatory Networks (GRNs) are complex systems of genes that interact with each other to control gene expression. Understanding GRNs is essential for understanding the underlying mechanisms of many biological processes. Machine Learning (ML) is a powerful tool for analyzing large-scale biological data and has been widely used in bioinformatics research. In our research, we use machine learning techniques in a novel way. Agents are not viewed as classic predictors capable of predicting the time sequence of variables, but are considered as specific units that have learned from data the capability to regulate the gene expression of each single gene. An agent regulates the expression of the corresponding gene based on the gene expression of other genes in the environment. The possibility to have different architectures for agents is crucial for being able to replicate the behavior of actual gene expression simulation.

We will now discuss four crucial concepts that have emerged in our paper: the capacity to forecast gene expression over time, given the initial gene expression level at Time 0; the validation of the results obtained with a perturbation applied to a gene; the differences in performance between artificial and real datasets; the computational cost.

Finally, we will present our future work and discuss how we can improve this approach.

### 6.1. Gene Expression Forecasting

In the previous sections, we introduced a novel approach to modeling gene expression using an artificial environmental setting. Our primary objective has always been to create a framework that can accurately replicate gene expression behavior, thereby simplifying the process of conducting experiments in the real world and providing researchers with more-precise data. To validate our methodology, we conducted several experiments, the results of which are presented in Section 5. Our simulation experiments demonstrated that our model can accurately simulate the expression over time of a group of genes. The reliability of the environment was high for all datasets considered, as evidenced by Figure 6 and Figure 7, which were extracted from two datasets as examples. These figures demonstrate that our model can reliably forecast gene expression over time, given the initial gene expression as the input.

### 6.2. Gene Regulation Validation

However, simply forecasting gene expression is not enough to determine the capacity of our AES to replicate gene behavior. Therefore, in Section 5.3, we aimed to demonstrate that introducing a perturbation to a gene causes other genes that are highly correlated with it to alter their gene expression in a manner consistent with the expected behavior. These alterations are presented in Table 6. It should be noted, however, that two incorrect relations were identified: in the first case, a gene was classified as a regulator (polB on lexA), resulting in a false positive; in the second case, a relation between the regulator and the regulated gene was missing (lexA and uvrA), resulting in a false negative. Nevertheless, this information, such as that presented in Table 6, can be highly valuable for biologists in determining gene regulatory networks. The data obtained can be evaluated by experts in the field to extract important knowledge about gene regulation. In fact, the analysis of a regulatory matrix may be more important than the extraction of a gene regulatory network itself, as it directly measures how pairs of genes interact with each other. It is worth noting that our model was able to extract this information with only a small amount of data. The SOS DNA Repair dataset contains four experiments, each with 50 observations. Therefore, it is highly probable that, with a higher number of observations, we would obtain results that are much more similar to reality.

### 6.3. Artificial vs. Real Datasets

The last step involves inferring a gene regulatory network, and it is worth noting that our methodology performs better with real datasets than with artificial ones. In fact, when combined with SOS DNA Repair, we achieved a high AUC that surpassed the state-of-the-art method, as reported in [11]. This is not surprising, as models trained on real datasets are more likely to capture the complexity and variability of real biological systems. Real datasets are inherently more complex, noisy, and biologically relevant, and they may contain all the relevant features that are necessary for accurate modeling. In contrast, artificial datasets are often designed to test specific aspects of a model’s performance and may not include all the relevant features present in real biological systems [5]. As a result, models trained on artificial datasets may be overfit and perform poorly when applied to real biological data. Therefore, it is crucial to use real datasets to train gene regulatory network models to ensure accurate predictions and capture the underlying biology.

### 6.4. Computational Cost

The issue of computational costs must necessarily be addressed when creating an AES from a gene expression time-series. This process is more time-consuming than existing methodologies, as it involves exploring several possible configurations of the AES and discarding those that do not perform well. The computational cost of our methodology mainly depends on two factors: neural network training (Section 4.1.2) and the running time of the optimization algorithm (Section 4.1.4). The training of neural networks can be computationally expensive, especially when dealing with large datasets and complex models. Additionally, it is not possible to estimate the time needed to train a neural network with absolute certainty due to the variability of the data and the model. Analogously, the running time of the genetic algorithm can also vary depending on various factors, such as the solution size and the number of neural network types available for each agent. To address these computational challenges, we adopted several techniques such as multiprocessing (training each neural network from an agent in parallel) and caching memory (training an agent’s neural network architecture once and using it for subsequent configurations of the AES). By implementing these techniques, the time required to create an AES can be significantly reduced. Obviously, as the number of genes increases, the computation time also increases; however, once an AES is created for a specific dataset, the process of extracting a regulatory matrix and inferring gene regulation is faster than other methods, as it involves computing simple equations (reported in Section 4.2.3) and it is also deterministic. Although creating an AES is computationally expensive, it is still less expensive than conducting a real experiment in a laboratory.

### 6.5. Future Work

In this work, we presented a novel approach aimed at providing a new solution to gene expression regulation and similar real-world problems. However, the proposed methodology is still in its initial phases, and there is much room for improvement. The extraction of gene regulatory networks from regulatory matrices may not work for all types of networks under study. Nevertheless, the main focus of this work is not to infer gene regulatory networks, but to extract a regulatory matrix that can be studied by experts in the field and potentially discover new knowledge about gene interactions. As the number of genes increases, there is a need for a method that can process the regulatory matrix autonomously, and we are working on this.

In addition, we are also working to speed up the training process of our model. The idea is to integrate more-advanced training techniques, such as the concept of continuous learning, which can speed up the training process of each agent related to genes. Additionally, having an artificial environmental setting capable of replicating real-world behavior can be very useful, and we are, therefore, focusing on improving its reliability as well. Using a different time step in the matrix state can yield different results, and therefore, we are analyzing the trade-off between reliability, overfitting, and computational cost.

As can be seen, our method performs poorly with artificial datasets compared to real datasets. Therefore, we are working to extract and test real gene expression from organisms’ cells containing thousands of genes and use the methodology to simulate gene regulation behavior. These aspects will be analyzed in detail in future works.

## Figures and Tables

**Table 1:** Gene expression time-series datasets.

Name	Description	Source
DREAM3	Artificial dataset generated by GeneNetWeaver. It contains experiments with 10 and 50 genes. Each experiment has 21 observations.	[7]
DREAM4	Artificial dataset generated by GeneNetWeaver. It contains experiments with 10 and 100 genes. Each experiment has 21 observations.	[11]
SOS DNA Repair	Real dataset obtained by observing how the gene expression of 8 genes evolves over time.	[8]

**Table 2:** Neural network models and their architecture used to implement agents. The hyperparameters of the layers marked with * are chosen by a hyperparameter-tuning algorithm. For a comprehensive explanation of the hyperparameter values for each layer and the available activation functions, see Appendix A.

Neural Networks	Layers
Fully Connected Neural Network (FCNN)	• Input Layer (Input Size n×m) • Fully Connected Layer * • Activation Function * • Dropout Layer * • Fully Connected Layer * • Activation Function * • Fully Connected Layer * • Activation Function * • Fully Connected Layer (Output Size 1) • Output Layer
Convolutional Neural Network (CNN)	• Input Layer (Input Size n×m) • Convolutional Layer 1D (Filter 64, Kernel 2) • Activation Function * • Max Pooling 1D (Pool Size 2) • Fully Connected Layer * • Activation Function * • Dropout Layer * • Fully Connected Layer * • Activation Function * • Fully Connected Layer (Output Size 1) • Output Layer
Recurrent Neural Network (RNN)	• Input Layer (Input Size n×m) • Long-Short-Term-Memory Units * • Activation Function * • Fully Connected Layer * • Activation Function * • Dropout Layer * • Fully Connected Layer * • Activation Function * • Fully Connected Layer (Output Size 1) • Output Layer

**Table 3:** Values for reliability and error.

ID	Name	Genes	${\mathrm{Err}}_{\mathrm{MSE}}$	${\mathrm{Err}}_{\mathrm{MAE}}$	$R_{\rho }$ (%)	$R_{\eta }$ (%)
1	DREAM3_Ecoli_size10_1	10	$1.318\times 10^{-3}$	$1.554\times 10^{-2}$	92.615	99.324
2	DREAM3_Ecoli_size10_2	10	$1.108\times 10^{-3}$	$1.351\times 10^{-2}$	89.568	99.229
3	DREAM3_Ecoli_size10_3	10	$8.701\times 10^{-4}$	$1.299\times 10^{-2}$	88.890	99.808
4	DREAM3_Ecoli_size50_1	50	$1.710\times 10^{-3}$	$1.796\times 10^{-2}$	85.484	99.343
5	DREAM3_Ecoli_size50_2	50	$1.511\times 10^{-3}$	$1.671\times 10^{-2}$	85.256	98.945
6	DREAM3_Ecoli_size50_3	50	$1.707\times 10^{-3}$	$1.720\times 10^{-2}$	84.315	98.302
7	DREAM4_insilico_size10_1	10	$1.545\times 10^{-3}$	$1.773\times 10^{-2}$	94.567	99.700
8	DREAM4_insilico_size10_2	10	$1.841\times 10^{-3}$	$1.961\times 10^{-2}$	93.248	99.683
9	DREAM4_insilico_size10_3	10	$1.356\times 10^{-3}$	$1.780\times 10^{-2}$	95.025	99.734
10	DREAM4_insilico_size10_4	10	$1.165\times 10^{-3}$	$1.542\times 10^{-2}$	96.231	99.710
11	DREAM4_insilico_size10_5	10	$1.811\times 10^{-3}$	$2.195\times 10^{-2}$	96.101	99.221
12	DREAM4_insilico_size100_1	100	$2.581\times 10^{-3}$	$2.160\times 10^{-2}$	83.616	98.840
13	DREAM4_insilico_size100_2	100	$2.547\times 10^{-3}$	$2.157\times 10^{-2}$	83.432	99.386
14	DREAM4_insilico_size100_3	100	$3.164\times 10^{-3}$	$2.518\times 10^{-2}$	81.984	98.877
15	DREAM4_insilico_size100_4	100	$2.311\times 10^{-3}$	$2.127\times 10^{-2}$	84.945	99.374
16	DREAM4_insilico_size100_5	100	$1.935\times 10^{-3}$	$1.918\times 10^{-2}$	83.300	99.182
17	SOS DNA Repair	8	$4.440\times 10^{-4}$	$1.181\times 10^{-2}$	99.126	99.800

**Table 4:** Comparing the performance of optimized and non-optimized AES by using the SOS DNA Repair dataset. Bold highlights the best results.

Agent Type	Hyperparameter Optimization	${\mathrm{Err}}_{\mathrm{MSE}}$	${\mathrm{Err}}_{\mathrm{MAE}}$	$R_{\rho }$ (%)	$R_{\eta }$ (%)
FCNN	Yes	$7.958\times 10^{-3}$	$5.461\times 10^{-2}$	90.467	93.204
FCNN	No	$1.038\times 10^{-2}$	$6.130\times 10^{-2}$	89.593	91.199
CNN	Yes	$5.228\times 10^{-3}$	$4.564\times 10^{-2}$	92.434	95.476
CNN	No	$5.173\times 10^{-3}$	$4.718\times 10^{-2}$	92.124	95.409
RNN	Yes	$4.906\times 10^{-3}$	$4.400\times 10^{-2}$	92.062	95.459
RNN	No	$1.117\times 10^{-2}$	$5.949\times 10^{-2}$	75.739	90.852
**Mixed**	**Yes**	$4.440\times 10^{-4}$	$1.181\times 10^{-2}$	99.126	99.800

**Table 5:** List of the neural network architecture assigned to each agent in order to maximize the AES reliability. The dataset considered is SOS DNA Repair.

Gene Names	uvrD	lexA	umuDC	recA	uvrA	uvrY	ruvA	polB
**Agent Types**	RNN	RNN	CNN	RNN	CNN	CNN	RNN	RNN

**Table 6:** Regulatory matrix of SOS DNA repair. The second row contains the regulatory value obtained from a perturbation of the gene lexA. All the values reported are the absolute value of the regulatory value computed according to Equation (16). Green cells indicate values that are consistent with real-world observations, while red cells represent false relationships.

	uvrD	lexA	umuDC	recA	uvrA	polB
uvrD	$2.4626\times 10^{-3}$	$1.5913\times 10^{-2}$	$5.8119\times 10^{-2}$	$2.0963\times 10^{-2}$	$2.6780\times 10^{-2}$	$1.1307\times 10^{-2}$
**lexA**	$3.4152\times 10^{-1}$	$1.7021\times 10^{-2}$	$1.5025\times 10^{-1}$	$2.3371\times 10^{-1}$	$8.6508\times 10^{-3}$	$9.4471\times 10^{-1}$
umuDC	$1.6370\times 10^{-1}$	$9.0818\times 10^{-3}$	$7.3554\times 10^{-4}$	$6.2446\times 10^{-2}$	$1.8608\times 10^{-2}$	$2.5642\times 10^{-1}$
**recA**	$1.0145\times 10^{-1}$	$4.7732\times 10^{-2}$	$7.8055\times 10^{-2}$	$1.4553\times 10^{-4}$	$1.9012\times 10^{-3}$	$1.9558\times 10^{-1}$
uvrA	$1.2966\times 10^{-1}$	$2.8761\times 10^{-2}$	$9.7740\times 10^{-2}$	$2.2303\times 10^{-2}$	$2.0379\times 10^{-3}$	$1.6112\times 10^{-1}$
polB	$3.5621\times 10^{-2}$	$6.4840\times 10^{-2}$	$4.2384\times 10^{-2}$	$1.8242\times 10^{-2}$	$1.8230\times 10^{-3}$	$5.6774\times 10^{-3}$

## Abbreviations

The following abbreviations are used in this manuscript:

AES	Artificial Environmental Settings
GRN	Gene Regulatory Network
ODE	Ordinary Differential Equation
MLP	Multi-Layer Perceptron
RNN	Recurrent Neural Network
CNN	Convolutional Neural Network
MSE	Mean-Squared Error
MAE	Mean Absolute Error
*n*	Number of genes
*b*	Number of observations
*m*	Number of steps considered in the state matrix
*k*	Number of experiments
$A_{i}$	A generic agent related to the *i*-th gene
$h\left(\cdot \right)$	Agent function
x	State of the environment
X	State matrix of the environment
$R_{\rho }$	Environment reliability based on Pearson correlation
$R_{\eta }$	Environment reliability based on cosine similarity
$Err_{MSE}$	Environment error based on MSE
$Err_{MAE}$	Environment error based on MAE
$\varphi \left(\cdot \right)$	Perturbation function
$\varphi_{d}$	Duration of a perturbation
$\Delta t_{r}$	Perturbation instability interval
$G_{i_{\max}}$	Maximum gene expression level observed for the *i*-th gene in the dataset

## Appendix A. Neural Network Hyperparameter Optimization

Neural network hyperparameter optimization is a crucial aspect of deep learning, which involves the selection of optimal hyperparameters for a given neural network architecture. Hyperparameters are parameters that are not learned during the training process, but are set prior to training and can significantly impact the performance of the model. Examples of hyperparameters include the learning rate, batch size, number of hidden layers, and activation functions.

The process of hyperparameter optimization involves searching for the best combination of hyperparameters that results in the highest accuracy or lowest error rate on a given dataset. This process can be time-consuming and computationally expensive, as it often involves training and evaluating multiple models with different hyperparameter settings. Table A1 presents a comprehensive list of hyperparameter values that can be assigned to each type of layer in a neural network, along with the training settings.

**Table A1:** Hyperparameters of a neural network with their possible values.

(a) Hyperparameters of Neural Network Layers		
**Layer Type**	**Hyperparameter**	**Possible Values**
Fully Connected Layer	Output Size	From 50 to 1000
Dropout Layer	Dropout Rate	From 0 to 1
Activation Function	Non-Linear Function Name	relu, Linear, elu, tanh, gelu, selu
LSTM Layer	Number of LSTM Hidden Units	From 2 to 1024
(**b**) **Training Settings**		
**Parameter Name**	**Description**	**Possible Values**
InitialLearnRate	It determines the initial learning rate of the neural network during training.	From 0.001 to 0.1
MaxEpochs	It determines the maximum number of epochs (iterations) that the neural network will be trained for.	From 50 to 500
Shuffle	It determines whether the training data are shuffled before each epoch.	Enable, disable
Optimizer	It determines the optimization algorithm used to update the weights of the neural network during training	Stochastic Gradient Descent (SGD), Adam

There are several approaches to hyperparameter optimization, including manual tuning, grid search, random search, and Bayesian optimization. We used a genetic algorithm for hyperparameter optimization. It starts with a population of randomly generated neural network hyperparameters and evaluates their performance on a validation set. The hyperparameters with the best performance are selected for reproduction, and their genetic material is combined to create a new population of hyperparameters. This process is repeated for several generations until the optimal set of hyperparameters is found.
